# Empirical Comparison of Simple Sequence Repeats and Single Nucleotide Polymorphisms in Assessment of Maize Diversity and Relatedness

**DOI:** 10.1371/journal.pone.0001367

**Published:** 2007-12-26

**Authors:** Martha T. Hamblin, Marilyn L. Warburton, Edward S. Buckler

**Affiliations:** 1 Institute for Genomic Diversity, Cornell University, Ithaca, New York, United States of America; 2 Centro Internacional de Mejoramiento de Maíz y Trigo, El Batan, Mexico, Mexico; 3 United States Department of Agriculture-Agricultural Research Service, Ithaca, New York, United States of America; University of Uppsala, Sweden

## Abstract

While Simple Sequence Repeats (SSRs) are extremely useful genetic markers, recent advances in technology have produced a shift toward use of single nucleotide polymorphisms (SNPs). The different mutational properties of these two classes of markers result in differences in heterozygosities and allele frequencies that may have implications for their use in assessing relatedness and evaluation of genetic diversity. We compared analyses based on 89 SSRs (primarily dinucleotide repeats) to analyses based on 847 SNPs in individuals from the same 259 inbred maize lines, which had been chosen to represent the diversity available among current and historic lines used in breeding. The SSRs performed better at clustering germplasm into populations than did a set of 847 SNPs or 554 SNP haplotypes, and SSRs provided more resolution in measuring genetic distance based on allele-sharing. Except for closely related pairs of individuals, measures of distance based on SSRs were only weakly correlated with measures of distance based on SNPs. Our results suggest that 1) large numbers of SNP loci will be required to replace highly polymorphic SSRs in studies of diversity and relatedness and 2) relatedness among highly-diverged maize lines is difficult to measure accurately regardless of the marker system.

## Introduction

Until recently, Simple Sequence Repeats (SSRs), also called microsatellites, have been the genetic markers of choice, because they are economical to score, have high allelic diversity, and are usually selectively neutral [1]. Recent advances in technology, however, have produced a shift toward single nucleotide polymorphism (SNP) markers, particularly for model organisms with substantial genomic resources. Individual SNP markers, being biallelic, have lower information content than SSRs, but they occur at much higher density in the genome, are amenable to high-throughput methods such as genotyping arrays, and have lower genotyping error rates [2]–[5]. For an overview of many of the technical and statistical issues of SSR and SNP genotyping, see [6].

The different properties of SNP and SSR markers arise from inherent differences in their mutational processes as well as from biased sampling practices that intensify those differences. (In this paper, we use the term SNP to refer to a marker that is genotyped using high-throughput technology [3], not a polymorphism that has been scored in fully re-sequenced alleles.) Because nucleotide mutation rates are low (on the order of 10^−8^/bp/generation), the vast majority of SNPs are biallelic, and thus have a maximum heterozygosity of 0.5. Not only is the SSR mutational rate much higher (for dinucleotide repeats in maize, 5.2×10^−4^ to 1.1×10^−3^ /generation [7]), but the slippage process can create a virtually unlimited number of new alleles [8], [9]. Maximal heterozygosity can thus approach 1.0, and frequencies are often skewed toward rare alleles. While re-sequencing studies can detect large numbers of singleton SNPs in many population samples (depending on population history), the selection of informative SNPs for genotyping studies (i.e., ascertainment) ensures that most SNP marker alleles will segregate at intermediate frequency, exaggerating the difference in frequency spectrum that already exists between SNPs and SSRs [3], [4]. This ascertainment bias must be corrected if SNP data are to be used in estimation of population genetic parameters.

The great interest in mapping genes affecting human health and the early use of SNP markers in this field have produced several comparisons of SSR and SNP markers in family-based linkage studies, e.g. [10]. In these studies, as well as in linkage studies of experimental populations, the mutational processes of the markers are not of great consequence because only a small number of meioses are captured by the data. In contrast, marker choice might have a greater effect on inferences about relatedness and genetic diversity among groups of individuals that are recently diverged on an evolutionary timescale, but are not considered relatives, such as populations of cases and controls, or diverse germplasm collections within a cultivated species. At this level of divergence, SNP-based distances will be due almost entirely to drift, while SSR-based distances will also be due in part to mutation.

Most crop plant germplasm collections have been assembled on the basis of geographic and phenotypic diversity, with the goal of capturing as much functional genetic diversity as possible. To allow more efficient exploitation of germplasm collections, core subsets are typically assembled by various criteria, e.g. [11], [12], and eventually characterized with presumably neutral molecular markers. Once the accessions have been scored with a common set of markers, estimation of genetic relationships among accessions provides information that is critical for choice of breeding material and for design of experimental crosses. In maize, for example, prediction of productive hybrid combinations and assignment of lines to heterotic groups has been based on the hypothesis that heterosis of hybrids increases monotonically with increasing genetic distance of the parents [13]–[15].

Inference of ancestry among unrelated individuals is also important because of its influence on the accuracy of population-based methods for genetic mapping of complex traits: to prevent spurious results, ancestry must be assessed and accounted for in the analyses. The most widely-used approach is the model-based method of Pritchard *et al.* [16], which assigns individuals membership in a population based on their alleles at a large number of genome-wide markers. Using this method, Rosenberg *et al.* [17] found that dinucleotide repeat SSRs in humans are five to eight times as informative as random SNPs, a result that was confirmed by Liu *et al.* [18].

Given the trend toward increased use of SNP markers, it is of interest to compare the performance of these two types of markers for characterization of the same set of individuals in a model crop plant, maize. In a recent study, Jones *et al.* [19] collected data for 80 SSRs and 187 SNPs in the same set of 58 inbred maize lines representing a subset of maize diversity derived from temperate Northern Flint and Southern Dent landraces. They demonstrated the technical advantages of SNPs over SSRs with respect to data quality, and found that measures of genetic distance based on SSRs, SNPs, and SNP haplotypes were not significantly correlated unless the inbreds were related by pedigree.

We were interested in exploring further, with a larger data set, how evaluation of population structure and measures of genetic distance are affected by choice of marker type, and what properties of the markers are responsible for differences in their usefulness. In this paper, we compare analyses based on 89 SSRs (primarily dinucleotide repeats) to analyses based on 847 SNPs in individuals from the same 259 inbred maize lines. These lines represent the widest genetic diversity available among current and historic lines used in breeding [20], including tropical and semi-tropical lines. We ask to what extent these two classes of markers provide concordant information about the structure of populations and the relationships among individuals. We also test whether any SNPs are more strongly differentiated than would be expected by chance, suggesting a history of positive selection.

## Results

### Summary statistics

**Figure 1:**
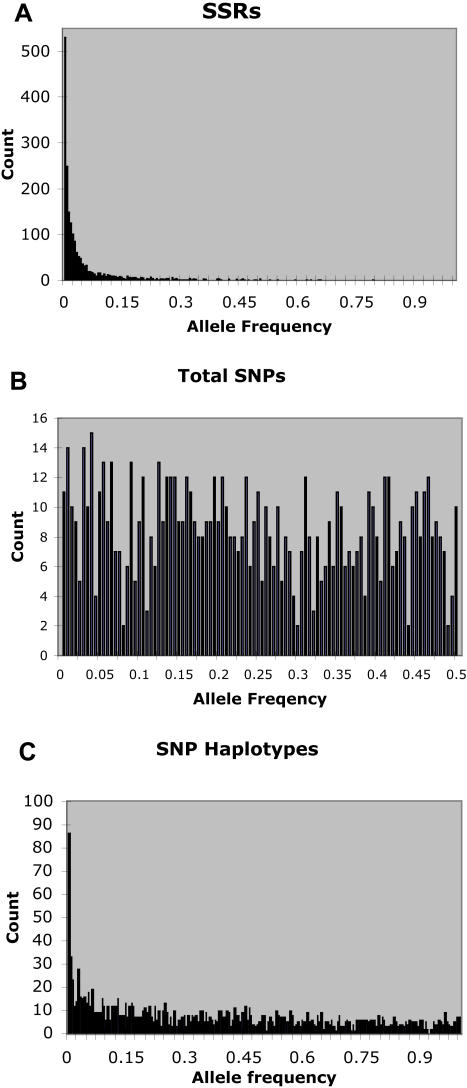
Allele frequency spectra for different classes of markers. Note that the scale on the x axis is different for total SNPs, because only this class is biallelic.

As expected, the allelic diversities and frequency spectra were very different for these data sets (Table 1, Figure 1). Due to the high rate of SSR mutation, this fairly modest number of SSR loci had a very large number of alleles, most at low frequencies in the population, and a large number of singletons. In contrast, the SNP loci showed large numbers of intermediate frequency alleles. Clearly, this was a consequence of ascertainment bias, since only SNPs that were discovered in a small set of lines were included in the study. Such bias is inherent to all SNP-based studies.

**Table 1:** Properties of different marker sets.

Marker	# Loci	# Alleles	# Singletons	*H_e_*
SSR	89	1872	540	0.801
SNP	847	1694	11	0.319
SNP haplotype	554	1480	82	0.387

To make greater use of the information content of the SNP data, we constructed haplotypes for those loci where more than one SNP had been scored from the same amplicon (see Methods). The 554 loci in the “SNP Haplotypes” set had fewer total alleles than the 847 single SNPs because, at most loci, not all gametic types were observed (Table 2) and because more than half the loci were still single SNPs. However, expected heterozygosity was increased by about 20%, and the frequency spectrum shifted slightly toward rarer alleles, including substantially more singletons (Table 1).

**Table 2:** Number of haplotypes observed in multi-SNP amplicons.

SNPs/locus (# amplicons)	2 alleles	3 alleles	4 alleles	5 alleles	6 alleles
1 (282)	282	-	-	-	-
2 (251)	16	147	88	-	-
3 (21)	1	6	5	3	6

### Assessment of population structure

**Figure 2:**
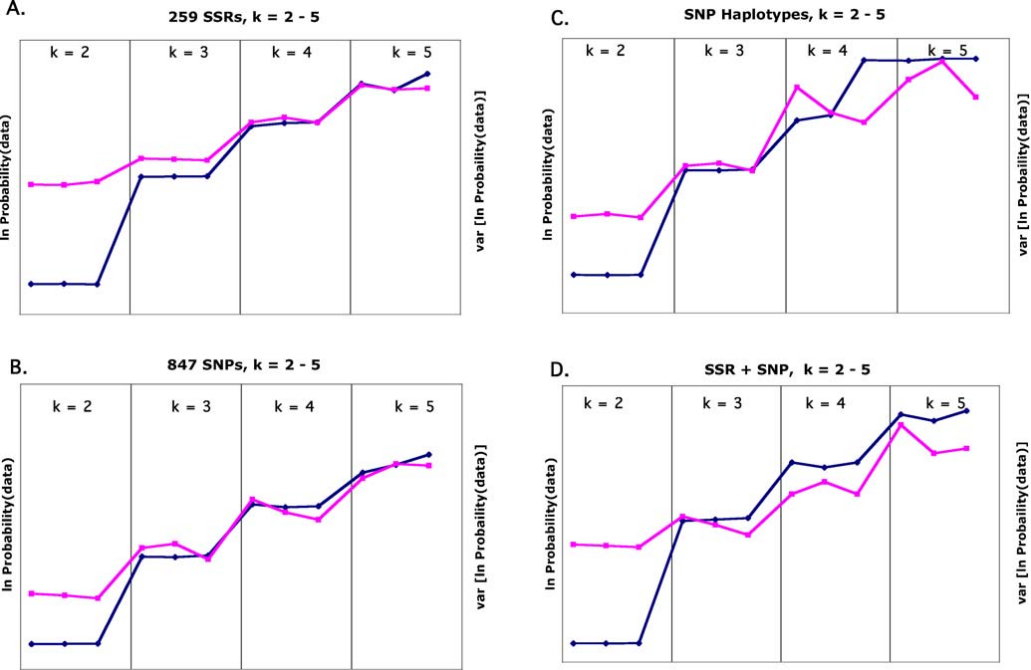
Estimated ln(probability of the data) and Var[ln(probability of the data)] for k from 2 to 5. Values are from STRUCTURE run three times at each value of k, using A) 89 SSRs; B) 847 SNPs; C) 554 SNP haplotypes; D) 89 SSRs+847 SNPs. The blue diamonds are ln(probability of the data) and the pink squares are var[ln(probability of the data)].

Using SSR variation, Liu *et al.* [20] showed that maize diversity is best described as belonging to three population groups: Stiff Stalk (SS), Non-Stiff Stalk (NSS), and Tropical-Semitropical (TS). We used the same approach as Liu, namely the model-based method of Pritchard *et al.* [16], to compare the SSR and SNP marker sets' ability to detect population structure and assign individuals to populations (see Methods). Figure 2 shows that, for all data sets, likelihood increased most when k (the number of populations in the model) was increased from two to three; results were very consistent across runs with k = 3 but became less consistent at higher values of k. Interestingly, the percent of individuals assigned to populations did not continue to increase with k, as might be expected: maximal assignment occurred at k = 2 , 3, or 4, depending on the markers used (Table 3), though many of the differences in percent assignment were small. The most dramatic difference was between k = 2 and k = 3 for the SNP+SSR data set: almost 20% more individuals were assigned under the model with k = 3. Strikingly, all assignment percentages for this data set were lower than for the SSR data set alone.

**Table 3:** Percent population assignment based on different marker sets.

Marker	k = 2	k = 3	k = 4	k = 5
SSR	**78.0**	76.4	66.8	69.1
SNP	40.1	52.5	**55.2**	50.6
SNP Haplotype	41.3	**55.2**	52.5	51.7
SNP+SSR	48.3	**67.6**	62.3	59.8

Each value gives the percent of individuals that had ≥0.8 membership in a population under that model with that data set (see Methods).

For all values of k tested, it is clear that the SNP data did not contain sufficient information to resolve all the relationships that were detected by SSR variation, resulting in a lower percentage of individuals that could be assigned to a population. This was not due simply to the higher allele number, since a reduced data set of 80 SSRs with 1694 alleles (the same number as the SNP data set) resulted in the same percent assignment as the full data set (data not shown).

**Figure 3:**
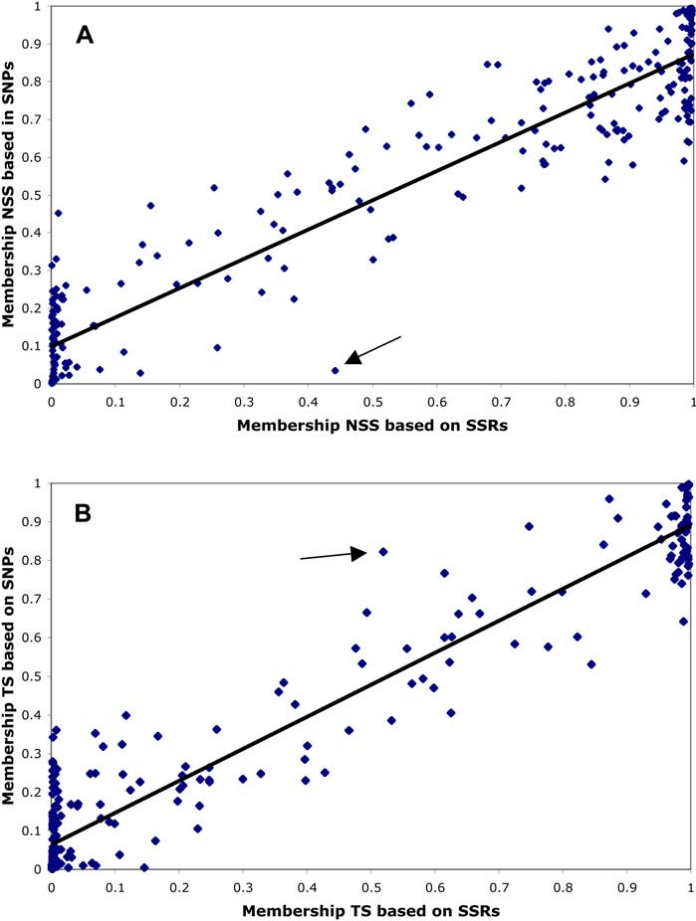
Comparison of membership in population clusters based on marker class. Each point represents one individual's proportion of ancestry in the NSS (top panel) or TS (bottom panel) cluster, based on SSR (x axis) or SNP (y axis) data assuming three populations. The arrows indicate line F2834T (see text).

The assignment of individuals to populations was very consistent among marker types, for those individuals that were assigned. Assuming three populations as described by Liu *et al.* [20], we plotted the relationship between membership in the NSS and TS populations based on SNPs and membership based on SSRs (Figure 3). The correlations were strong (R^2^ = 0.88 and 0.93, respectively), but there was clearly much more spread along the y axes (SNPs) than along the x axes (SSRs): 116 lines had NSS membership between 0.2 and 0.8 based on SNPs, as opposed to only 58 lines that fell in that range based on SSRs. In no case was an individual assigned to a different population based on different marker information. In a small number of cases, SNP data resulted in assignments for individuals that were unassigned using SSR data. One such example is line F2834T, indicated by an arrow in each panel of Figure 3. Based on SNP data, F2834T had 82% of its ancestry in the TS cluster, while, based on SSR data, it had only 52% of its ancestry in that cluster and was thus classified as Mixed.

### Distance measures

**Figure 4:**
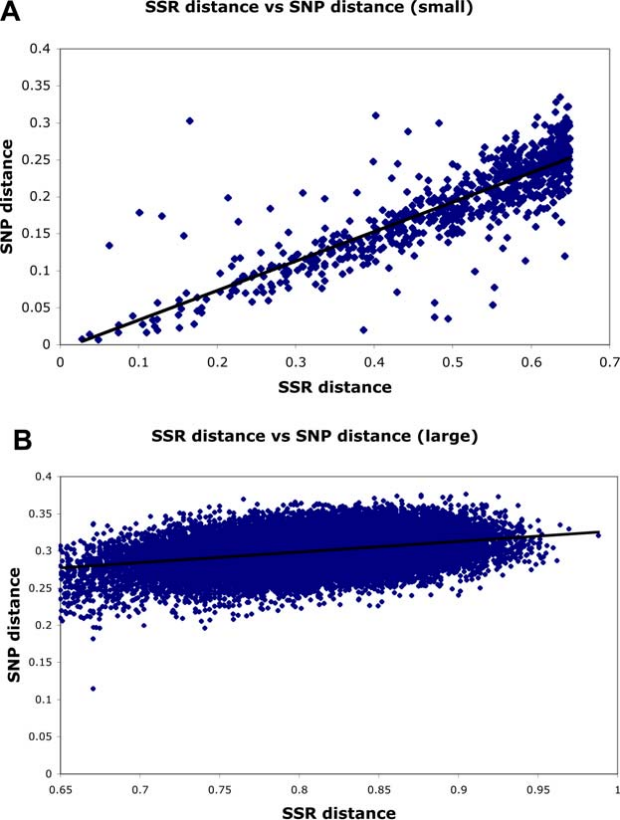
Correlation between genetic distance based on SSRs and SNPs. Each point represents the genetic distance between a pair of individuals, based on sharing of SSR (x axis) or SNP (y axis) alleles. The top panel shows the relationship for pairs of individuals whose SSR distance is <0.65; the bottom panel shows the relationship for pairs of individuals whose SSR distance is ≥0.65.

Distance matrices based on allele sharing were constructed for all pairs of individuals using either SSR data or total SNP data, and the relationship between the distance matrices was plotted (Figure 4). For the small percentage of individual pairs that were closely related (SSR Distance <0.65), there was a strong correlation between the distances for the two marker types (R^2^ = 0.73, Fig 4 top). However, the vast majority (97.7%) of SSR-based distances were large (≥0.65), and for those comparisons the correlation with SNP-based distance was modest (R^2^ = 0.11). The lower panel of Figure 4 shows that, while SSR-based distances varied from 0.65 to over 0.95, the SNPs provided poor resolution in this group, with distances that varied only from about 0.2 to 0.35. While comparisons at the individual level were not well-correlated across marker types, this was not the case for population-level comparisons: the correlation between allele-sharing distance among populations was >0.99 (Mantel test).

Given that the SSR data set had a much larger proportion of low frequency alleles, we tested whether this difference was responsible for the decrease in resolution provided by the SNP data set. To do this, we divided the SNP data into five frequency classes, calculated all pairwise distances based on each class, and calculated the correlation between those distances and the SSR-based distances. Although the results were slightly confounded by sample size, Table 4 shows that the intermediate frequency SNPs were better able to resolve shorter distances, but that the low frequency SNPs contributed most of the resolution when distances were larger. Distances based on the SNPs at intermediate frequency are the best correlated with the distances based on SSRs, even though the SSR data set was dominated by rare alleles.

**Table 4:** Correlation between genetic distance for SSRs and SNPs of different frequency classes.

SNP Class	# loci	Minor allele freq	R^2^ between SNP distance and		
			SSR dist^a^	SSR dist (<0.65)^b^	SSR dist (≥0.65)^b^
1	185	p≤0.10	0.109	0.175	0.072
2	188	0.10<p≤0.2	0.160	0.590	0.046
3	164	0.20<p≤0.3	0.161	0.652	0.020
4	149	0.30<p≤0.4	0.136	0.596	0.013
5	162	0.40<p≤0.5	0.190	0.645	0.023
123	536	p≤0.30	0.295	0.678	0.100
total	847	0<p<1	0.394	0.726	0.114
haps	554	0<p<1	0.400	0.736	0.110

Measures of pairwise genetic distance among all individuals were calculated for different frequency classes of SNP alleles and for all SSRs. ^a^ The correlation between all pairwise distances based on a class of SNP loci vs based on SSRs. ^b^ The correlation between pairwise distances for the subset of pairs whose SSR distance is <0.65 or ≥0.65.

### Core sets

Liu *et al.* [20] found a total of 2039 SSR alleles in 260 inbred lines, and tested different sized “core sets” to see what fraction of the alleles could be captured. Because of the large number of rare alleles, 193 lines were needed to capture all 2039 alleles, and a set of 20 lines captured only 46% of the alleles. We repeated this analysis with our data sets, and found that a subset of 20 individuals captured almost all SNP alleles and over 90% of SNP haplotypes. Table 5 shows the percent of alleles captured by subsets of 20, 30, 40, and 50 for the various marker data sets. When we removed nine loci from the SSR data set so that it had exactly 1694 alleles (like the SNP data set), the percent alleles captured by subsets of these sizes remained the same (data not shown).

**Table 5:** Alleles captured by subsets of the data for different marker sets.

	SSRs		SNPs		SNP haplotypes		Sim SNPs SNM		Sim SNPs Bot1		Sim SNPs Bot2	
subset size	Allele #	% captured	Allele #	% captured	Allele #	% captured	Allele #	% captured	Allele #	% captured	Allele #	% captured
20	896	48	1659	98	1346	91	1414	83	1552	92	1493	88
30	1091	58	1684	99	1399	95	1486	88	1600	94	1558	92
40	1249	67	1685	99	1420	96	1530	90	1617	95	1596	94
50	1340	72	1693	100	1446	98	1563	92	1640	97	1624	96

Sim SNPs SNM, Bot1, and Bot2 represent core sets drawn from data generated by coalescent simulations under the standard neutral model and two bottleneck models (see Methods).

This comparison is somewhat misleading, however, because of the ascertainment of the SNPs in our data set. If all 259 lines had been fully re-sequenced, there would be more rare alleles to be captured. We therefore simulated a set of 847 unlinked SNPs in 259 individuals under the standard neutral model (SNM), producing the full frequency spectrum of variation. As expected, the percent of alleles captured in a small core set dropped substantially (from 98% to 83% for a subset of 20), but was still much higher than the percent of SSR alleles captured in a core set of the same size (Table 5). Furthermore, the SNM likely overestimates the number of rare alleles that would be observed in a fully re-sequenced maize data set, due to the effect of the domestication bottleneck. Since the exact parameters appropriate for simulating such a data set are unknown (see Discussion), we performed simulations under two reasonable bottleneck models for comparison to the SNM. As seen in Table 6, heterozygosity and the number of singletons in this data set were intermediate between the values observed in the ascertained SNP data set and under the SNM. The percent of alleles captured by these core sets was only slightly lower than was found for the ascertained data (Table 5), suggesting that we may not have missed a large number of rare SNP alleles.

**Table 6:** Properties of data sets simulating full resequencing.

Data set	*H_e_*	# Singletons
Observed (ascertained)	0.319	11
Simulated Standard Neutral	0.165	139
Simulated Bottleneck 1	0.255	100
Simulated Bottleneck 2	0.199	90

### Fst at individual loci

Overall *F_st_* was very similar regardless of marker set used (0.08–0.10), with *F_st_* based on SSRs being in the lower end of the range, as expected because of their higher heterozygosity. *F_st_* based on individual SNP loci, however, varied from 0 to 0.568; some of these differences in *F_st_* may be due to the action of natural or artificial selection at particular loci. To test this hypothesis, we used the FDIST2 program [21], which uses coalescent simulations to generate a neutral joint distribution of *F_st_* and *H_e_*. Loci that have an unusually large value of *F_st_*, given their heterozygosity, are candidates for having experienced selection.

We found that 32 SNPs, about 3.8% of the 847 loci tested, had p-values <0.05 (uncorrected for multiple tests), so there was little evidence for selection. P-values for two SNPs, both from the same locus (AY108077, in IBM2 Bin 6.04, with similarity to a calmodulin-binding heat-shock protein), were the only ones that were significant using a Bonferroni-corrected critical value of 0.05/847 = 0.000059. These two SNPs, whose alleles are in complete linkage disequilbrium (i.e., r^2^ = 1), showed a pattern that was very common among the SNPs with higher *F_st_*: the NSS and TS populations had the same allele at very high frequency, while the SS population had the alternate allele at a very high frequency. Usually the allele at high frequency in NSS and TS was also at high frequency in Mexican samples that included teosintes (these frequencies can be viewed at www.panzea.org).

## Discussion

Molecular markers serve a variety of purposes in genetic analysis. In this study we compared the ability of SSR and SNP markers to assess relatedness in a population where divergence times are long enough that most individuals are considered unrelated and the true relationships are unknown. We found that a set of 89 highly polymorphic SSRs performed better at clustering germplasm into populations than did a set of 847 SNPs or 554 SNP haplotypes, and that SSRs provided more resolution in measuring genetic distance based on allele-sharing. We observed only slightly higher resolution when we converted SNPs to haplotypes, perhaps because fewer than half our SNPs could be converted.

These results are consistent with theoretical predictions [6], [22] and with other empirical studies, e.g. [17]. According to Laval *et al.* [22], (*k*-1) times more biallelic markers are needed to achieve the same genetic distance accuracy as a set of microsatellites with *k* alleles. In our case, the average number of alleles per SSR locus was about 20; thus we should need [(20–1) * 89] = 1691 SNP markers to achieve the same accuracy; we had almost exactly half that many SNP markers.

The difference in information content of SSRs and biallelic markers may also be due to differences in the frequency spectrum: SSR loci have many more low-frequency alleles than SNP loci, since at least half of all SNP alleles must be at high or intermediate frequency. This frequency difference is even greater when ascertained SNPs are genotyped.

### Population structure estimation

Population genetics theory predicts that “preferentially discovering SNPs with high heterozygosity leads to an underestimation of the magnitude of structure” [4]. Using SSR data and the model-based method of Pritchard *et al.* [16], Liu *et al.* [20] found that the 259 maize inbred lines in this study were best described as belonging to three populations, with about 22% of the lines having mixed ancestry. We found that our SNP data could support a model of three or four populations and were largely consistent with the results of Liu *et al.*, although, regardless of the number of populations in the model, far more individuals (44.8–47.5%) were classified as mixed using SNP data. It was surprising that, as k was increased, the likelihood increased but the percent assignment declined.

### Measures of genetic distance in maize

Our analysis showed that most maize lines in a diverse sample are separated by a large genetic distance and, consistent with the results of Jones *et al.* [19], that measures of distance based on different markers were well-correlated only for the small subset of individuals that were closely related. Other studies of tropical maize, which is extremely diverse, have found that SSR variation does not provide evidence of population structure other than among individuals closely related by pedigree [23], [24]. Collectively, these results suggest that relatedness among highly-diverged maize lines is difficult to measure accurately regardless of the marker system. This may explain the observation that only intragroup crosses show a correlation between parental genetic distance and midparent heterosis [25]. Individuals within groups have smaller genetic distances that can be more accurately assessed, while intergroup comparisons are in the range where resolution is poor (Fig 3 top).

Yu *et al.* [26] used essentially the same data set as this one to test the sensitivity of relatedness estimation to marker number, as assessed by the power to detect QTL in mixed-model association mapping. Contrary to our results, they found that “the whole set of 89 SSR markers provided roughly the same amount of information as did the whole set of 912 SNP markers for relatedness construction.” This apparent discrepancy is probably due to the fact that, in the calculation of the kinship matrix, kinship estimates for individuals who were less related than average were all set to zero. Thus no attempt was made to estimate the larger genetic distances. Given this strategy, the difference in resolution was not of great practical importance. However, this may not be the optimal strategy. Accurate estimate of identity-by-state for thousands of markers may provide even greater power and control of type I errors.

### Implications for germplasm conservation

The development of core sets is often described in terms of the fraction of SSR alleles retained in subsets of the collection. An implicit assumption is that the presence of unique SSR alleles in an individual or population is an indicator of the presence of unique functional variation. As stated in Laborda *et al.* [27], their goal was to “expose useful diversity for breeding purposes” by maximizing allelic diversity at markers. Similarly, Lockwood et al [28] state: “Sampling for allelic richness is important for conservation and in the development of genetic resource collections.” It is well known that most SSR variation is neutral, and that functional variation is much more likely to be associated with SNPs and indels in and around genes. Since a small sample of alleles captures most of the SNP variation segregating in a population, is it truly important to capture rare SSR alleles in germplasm collections?

We have shown that 98% of the (ascertained) SNP allelic variation in a sample of 259 diverse lines can be captured in a set of 20 lines, as opposed to only 48% of the SSR alleles. How much of this difference is due to the loss of rare SNP alleles due to ascertainment? Data sets simulating fully re-sequenced data showed that rare SNP alleles are indeed missed, however, because maize is not a population at equilibrium, it is not clear how many singletons would have been found by complete re-sequencing of these inbred lines. In a mix of landraces and inbreds, Tenaillon et al [29] found an average D [30] of about 0.1, and reported that average D was higher in the inbreds alone, indicating a loss of rare alleles. The bottleneck models reported in Tables 5 and 6 are suggestive but do not reproduce the actual frequency spectrum in maize, so these results must be interpreted with caution. In any case, the number of lines required to capture all SNP alleles even in the non-bottlenecked population is far smaller than that required to capture all rare SSR alleles. Generalizing beyond maize, the extent to which rare alleles are lost in SNP genotyping studies will vary among species, since different population histories will produce different frequency spectra of variation. Eventually, the dramatically lower cost of new sequencing technologies may allow for full re-sequencing to replace SNP genotyping, eliminating the problem of ascertainment altogether.

However, even if every SNP were observed and captured, SNP diversity at individual sites is unlikely to capture *all functional* variation, since different combinations of SNP alleles (both within and between loci) will give rise to haplotypes that may differ in their contributions to phenotypes of interest. SSR allelic diversity should be positively correlated with SNP haplotypic diversity for the simple reason that both attributes are functions of effective population size. (Due to their higher mutation rates, SSRs more accurately reflect effective population size in non-equilibrium populations.) As a proxy for larger effective population size, maximal SSR diversity ensures that novel combinations of functional SNP alleles are more likely to be captured in a sample. Note that this applies only at the genome-wide level: Payseur and Cutter [31] showed that SSR heterozygosity and nucleotide heterozygosity at linked sites are only weakly correlated under a number of different scenarios (maximum correlation <0.2), so we cannot expect that high SSR allelic diversity in a particular chromosomal region is indicative of high SNP or haplotype diversity as well. This suggests that capturing particular rare SSR alleles is not likely to capture rare functional variation at linked sites.

### General conclusions

While SNPs represent the latest technology, and have a number of technical advantages, there may be biological questions for which SSRs provide higher quality information. However, conclusions about the relative usefulness of SSRs and SNPs will vary depending on the specifics of the particular study. Because the SSR mutational process is very heterogeneous among loci [9], results of these kinds of comparisons may depend on the particular SSR loci, or class of loci (e.g., repeat-type), surveyed. In addition, the choice of marker type, and the number of loci needed, is a function of the evolutionary history of the populations being investigated [4], [9]. Extensive simulation studies are needed to understand how the interaction between mutational processes and population history affects population genetic inferences.

## Materials and Methods

### Plant material

Individuals from 259 maize inbred lines were used in this study: 33-16, 38-11, 4226, A188, A214N, A239, A272, A441-5, A554, A556, A6, A619, A632, A634, A635, A641, A654, A659, A661, A679, A680, A682, B10, B103, B104, B105, B109, B115, B14A, B164, B2, B37, B46, B52, B57, B64, B68, B73, B73Htrhm, B75, B76, B77, B79, B84, B97, C103, C123, C49A, CH701-30, CH9, CI187-2, CI21E, CI28A, CI31A, CI3A, CI64, CI66, CI.7, CI90C, CI91B, CM174, CM37, CM7, CML10, CML103, CML108, CML11, CML14, CML154Q, CML157Q, CML158Q, CML218, CML220, CML228, CML238, CML247, CML254, CML258, CML261, CML264, CML277, CML281, CML287, CML311, CML314, CML321, CML322, CML323, CML328, CML331, CML332, CML333, CML341, CML38, CML45, CML5, CML52, CML61, CML69, CML77, CML91, CML92, CMV3, CO106, CO125, CO255, DE1, DE_2, DE_3, DE811, E2558W, EP1, F2834T, F44, F6, F7, GA209, GT112, H105W, H49, H84, H91, H95, H99, Hi27, Hy, I137TN, I205, Ia5125, IDS91, K148, K4, K55, K64, Ki11, Ki14, Ki21, Ki3, Ki43, Ki44, Ky21, Ky226, Ky228, L317, L578, M14, M162W, M37W, MEF156-55-2, Mo17, Mo18W, Mo1W, Mo24W, Mo44, Mo45, Mo46, Mo47, MoG, Mp339, MS1334, MS153, MS71, Mt42, N192, N28Ht, N6, N7A, NC222, NC230, NC232, NC236, NC238, NC250, NC258, NC260, NC262, NC264, NC290A, NC294, NC296, NC296A, NC298, NC300, NC302, NC304, NC306, NC310, NC314, NC318, NC320, NC324, NC326, NC328, NC33, NC336, NC338, NC340, NC342, NC344, NC346, NC348, NC350, NC352, NC354, NC356, NC358, NC360, NC362, NC364, NC366, NC368, ND246, Oh40B, Oh43, Oh43E, Oh603, Oh7B, Os420, Pa762, Pa875, Pa880, Pa91, R109B, R168, R177, R229, R4, SC213R, SC357, SD44, T232, T234, T8, Tx303, Tx601, Tzi10, Tzi16, Tzi18, Tzi25, Tzi8, Tzi9, U267Y, Va102, Va14, Va17, Va22, Va26, Va35, Va59, Va85, Va99, VaW6, W117Ht, W153R, W182B, W22, W64A, WD, Wf9. These lines have been previously described [20].

### SSRs

Of the 89 SSRs used in this study, 76 are dinucleotide repeats, 5 are trinucleotide repeats, 7 are tetranucleotide repeats, and one is a pentanucleotide repeat. Analysis of a largely overlapping data set has been published before [20]. All 89 of the markers and 210 of the 259 lines in our study are common to the two data sets. Markers scored by Liu *et al.* [20] not used in this study are: bnlg1014, bnlg1189, bnlg1288, bnlg1520, bnlg1839, bnlg1931, bnlg2238, phi031, phi096, and phi116; information on all SSRs can be found in the MaizeGDB database (http://www.maizegdb.org/).

### SNPs

A collection of 913 SNPs that had been found to be polymorphic in a set of 14 maize inbred lines and 16 inbred teosintes [32] was scored in a set of 277 maize inbred lines [33]. They were designed from randomly selected genes out of the ∼10,000 maize ESTs in the MMP-DuPont set [34]. SNP assay development and scoring was performed by Genaissance Pharmaceuticals using the Sequenom MassARRAY™ System [35]. Replicated assays indicate that the genotyping error rate is ∼0.3%.

After exclusion of lines not common to the two data sets and markers with >20% missing data, the final data set consisted of 259 individuals scored for 847 SNPs and 89 SSRs. The SNPs were located in 563 PCR products throughout the genome, thus representing 563 loci. A list of the SNPs can be found in Dataset S1; information about each marker can be found at http://www.panzea.org/db/searches/webform/moldiversity_search.

Haplotypes were constructed when more than one SNP had been scored from a single amplicon. Because the individuals sampled came from inbred lines, the vast majority of SNP genotypes were homozygous, making haplotypes unambiguous. In cases where more than one SNP was heterozygous in an amplicon from one individual, haplotypes were inferred if only two haplotypes were observed in homozygous individuals (i.e., complete LD), otherwise they were treated as missing data.

### Data analysis

Summary statistics, genetic distances (allele sharing), core sets, and input files for STRUCTURE were obtained using PowerMarker [36]. To compare the assignment of individuals to NSS, SS, or TS populations using different marker sets, STRUCTURE [16] was run with k = 2, 3, 4, or 5 (i.e., assuming 2–5 populations) for each of four different data sets:

1) 89 SSRs.

2) 847 SNPs.

3) 554 SNPs or SNP haplotypes. SNPs from the same amplicon were grouped into haplotypes that were recoded as alleles. There were 251 2-SNP loci and 21 3-SNP loci, as well as 282 isolated SNPs. If the genotype of any SNP at a locus was missing in an individual, the entire locus was treated as missing data for that individual.

4) Data sets 1 and 2 combined.

For each value of k, STRUCTURE was run three times with burn-in and runs of 50,000 each. The admixture model was used, allele frequencies were assumed to be correlated, and alleles were assumed to be unlinked (an assumption that is violated by data sets 2 and 4). An individual was assigned to a population if it had ≥0.8 membership in that population.

For purposes of *F_st_* calculation, individuals were designated as SS, NSS, TS, or mixed, according to the results of STRUCTURE using the SSR data set with k = 3. The three groups identified by STRUCTURE were classified as Stiff Stalk (SS), Non-Stiff Stalk (NSS), or Tropical-Semitropical (TS) based on the membership of individuals that typify those groups [20].

### Tests for selection

We used the program FDIST2 [21] to test whether any of the values of *F_st_* observed for individual SNPs were unusually large, given average *F_st_* and locus-specific heterozygosity. Parameters for the simulations were: demes = 3, sampled populations = 3, sample size = 50 (following the recommendation of the FDIST2 documentation). Average *F_st_* in the simulations = 0.1, the average observed for the SNP data set.

### Simulations

Three data sets of 847 SNPs in 259 individuals were simulated using the program ms [37]. One was generated under the standard neutral model, the others were generated under two different bottleneck models, both of which are modifications from Wright *et al.* [32]. Bottleneck 1 used “./ms 259 847 -s 1 -eN .0013 .0076 -eN .00208 1”, which produced average D [30] of about 0.4, while Bottleneck 2 used “./ms 259 847 -s 1 -eN .0013 .05 -eN .00208 1”, which produced average D of about 0.2. In all cases, every SNP was sampled from a different locus, so that all SNPs were evolutionarily independent.

## Supporting Information

Dataset S1 SNP loci used in this study.
(0.03 MB DOC)
