# Supplementary material for: Empirical Comparison of Simple Sequence Repeats and Single Nucleotide Polymorphisms in Assessment of Maize Diversity and Relatedness
Source: PLoS One. 2007 Dec 26;2(12):e1367. doi: 10.1371/journal.pone.0001367 (PMC2137949; doi:10.1371/journal.pone.0001367)
Supplement: Dataset S1 — SNP loci used in this study. (0.03 MB DOC) [file pone.0001367.s001.doc]

**Dataset S1. SNP loci used in this study.** Information about these loci can be found at <http://www.panzea.org/db/searches/webform/moldiversity_search>

PZA00001.8 PZA00003.11 PZA00003.5 PZA00004.2 PZA00005.2 PZA00005.5 PZA00006.13 PZA00006.9 PZA00007.1 PZA00007.2 PZA00008.1 PZA00010.5 PZA00013.8 PZA00013.9 PZA00017.1 PZA00017.4 PZA00018.5 PZA00022.2 PZA00029.11 PZA00029.12 PZA00030.11 PZA00031.5 PZA00040.19 PZA00041.3 PZA00042.2 PZA00042.5 PZA00043.1 PZA00043.7 PZA00045.1 PZA00045.2 PZA00047.2 PZA00048.1 PZA00048.2 PZA00049.12 PZA00051.17 PZA00051.2 PZA00058.1 PZA00058.5 PZA00060.2 PZA00061.1 PZA00063.3 PZA00065.1 PZA00065.2 PZA00067.8 PZA00067.9 PZA00069.2 PZA00069.4 PZA00070.5 PZA00078.2 PZA00078.4 PZA00079.1 PZA00081.17 PZA00084.2 PZA00084.3 PZA00086.8 PZA00088.3 PZA00089.3 PZA00092.1 PZA00092.5 PZA00094.2 PZA00096.6 PZA00098.14 PZA00099.6 PZA00100.2 PZA00100.9 PZA00102.6 PZA00102.8 PZA00103.20 PZA00103.27 PZA00106.9 PZA00107.18 PZA00107.4 PZA00108.12 PZA00108.4 PZA00109.3 PZA00111.2 PZA00111.4 PZA00112.3 PZA00112.4 PZA00113.1 PZA00114.3 PZA00116.2 PZA00116.3 PZA00118.1 PZA00118.4 PZA00119.1 PZA00119.4 PZA00120.4 PZA00121.1 PZA00121.10 PZA00123.1 PZA00124.2 PZA00124.3 PZA00125.2 PZA00125.8 PZA00130.7 PZA00131.14 PZA00132.17 PZA00132.3 PZA00133.1 PZA00133.3 PZA00134.12 PZA00136.1 PZA00136.2 PZA00137.2 PZA00139.14 PZA00139.4 PZA00140.6 PZA00140.7 PZA00143.5 PZA00148.2 PZA00148.3 PZA00151.2 PZA00153.3 PZA00153.6 PZA00158.2 PZA00163.4 PZA00164.1 PZA00166.1 PZA00166.3 PZA00167.7 PZA00168.14 PZA00170.1 PZA00172.11 PZA00174.1 PZA00174.2 PZA00175.2 PZA00176.8 PZA00177.4 PZA00178.3 PZA00178.4 PZA00179.9 PZA00181.2 PZA00181.5 PZA00182.3 PZA00182.4 PZA00184.1 PZA00184.4 PZA00186.2 PZA00187.2 PZA00187.8 PZA00188.1 PZA00189.21 PZA00191.4 PZA00191.5 PZA00192.6 PZA00192.7 PZA00193.1 PZA00193.2 PZA00196.2 PZA00198.39 PZA00200.8 PZA00200.9 PZA00201.2 PZA00205.7 PZA00209.4 PZA00209.8 PZA00210.1 PZA00210.6 PZA00211.7 PZA00213.19 PZA00213.7 PZA00214.1 PZA00215.3 PZA00216.2 PZA00216.8 PZA00218.1 PZA00218.6 PZA00219.6 PZA00219.7 PZA00220.11 PZA00220.2 PZA00221.7 PZA00222.4 PZA00222.5 PZA00223.2 PZA00224.3 PZA00224.4 PZA00225.7 PZA00225.8 PZA00226.6 PZA00226.7 PZA00227.7 PZA00229.7 PZA00230.5 PZA00233.8 PZA00234.19 PZA00234.21 PZA00235.6 PZA00235.8 PZA00236.7 PZA00237.2 PZA00237.6 PZA00240.4 PZA00240.6 PZA00241.6 PZA00243.1 PZA00243.24 PZA00245.14 PZA00245.16 PZA00247.5 PZA00249.2 PZA00250.1 PZA00251.1 PZA00251.4 PZA00254.3 PZA00255.14 PZA00255.15 PZA00256.16 PZA00256.21 PZA00257.11 PZA00257.22 PZA00258.3 PZA00259.1 PZA00260.1 PZA00261.5 PZA00261.6 PZA00263.13 PZA00265.4 PZA00266.5 PZA00270.1 PZA00272.3 PZA00274.14 PZA00274.7 PZA00276.18 PZA00277.13 PZA00277.9 PZA00280.14 PZA00281.1 PZA00281.13 PZA00282.14 PZA00282.2 PZA00285.2 PZA00287.1 PZA00289.11 PZA00291.7 PZA00294.20 PZA00294.4 PZA00295.6 PZA00295.7 PZA00296.6 PZA00297.2 PZA00297.3 PZA00300.11 PZA00303.19 PZA00303.6 PZA00307.12 PZA00310.5 PZA00311.4 PZA00315.1 PZA00315.6 PZA00316.2 PZA00316.9 PZA00318.2 PZA00318.5 PZA00323.3 PZA00323.4 PZA00326.16 PZA00328.1 PZA00329.3 PZA00332.3 PZA00332.5 PZA00334.2 PZA00335.12 PZA00336.10 PZA00337.3 PZA00339.3 PZA00340.1 PZA00342.7 PZA00342.9 PZA00343.11 PZA00344.10 PZA00345.15 PZA00348.10 PZA00349.3 PZA00350.2 PZA00356.8 PZA00357.19 PZA00358.12 PZA00362.1 PZA00362.2 PZA00364.2 PZA00364.5 PZA00365.2 PZA00365.3 PZA00367.2 PZA00367.6 PZA00368.1 PZA00368.17 PZA00369.1 PZA00369.11 PZA00370.1 PZA00370.5 PZA00371.5 PZA00378.9 PZA00379.2 PZA00380.5 PZA00380.7 PZA00381.3 PZA00382.17 PZA00382.22 PZA00385.1 PZA00385.3 PZA00386.2 PZA00386.3 PZA00387.1 PZA00390.6 PZA00391.2 PZA00393.1 PZA00394.11 PZA00394.15 PZA00395.1 PZA00395.2 PZA00396.10 PZA00396.9 PZA00398.4 PZA00399.10 PZA00400.3 PZA00401.11 PZA00401.6 PZA00403.5 PZA00405.2 PZA00407.8 PZA00408.7 PZA00409.15 PZA00409.3 PZA00410.2 PZA00411.1 PZA00413.17 PZA00413.2 PZA00414.27 PZA00416.2 PZA00416.4 PZA00417.2 PZA00417.3 PZA00418.2 PZA00419.1 PZA00420.1 PZA00422.2 PZA00422.5 PZA00423.16 PZA00423.17 PZA00424.1 PZA00425.4 PZA00425.9 PZA00428.2 PZA00429.1 PZA00429.2 PZA00432.4 PZA00433.3 PZA00433.5 PZA00435.4 PZA00435.5 PZA00436.7 PZA00439.6 PZA00440.1 PZA00440.15 PZA00442.3 PZA00442.4 PZA00443.4 PZA00444.1 PZA00447.5 PZA00447.6 PZA00448.5 PZA00448.6 PZA00449.2 PZA00449.3 PZA00452.1 PZA00452.2 PZA00453.2 PZA00453.7 PZA00455.13 PZA00455.4 PZA00458.1 PZA00458.6 PZA00459.5 PZA00460.3 PZA00462.2 PZA00463.3 PZA00464.6 PZA00466.1 PZA00468.7 PZA00470.1 PZA00471.1 PZA00471.2 PZA00472.2 PZA00473.5 PZA00473.9 PZA00474.14 PZA00477.5 PZA00477.9 PZA00478.7 PZA00479.9 PZA00481.7 PZA00482.10 PZA00484.5 PZA00484.6 PZA00485.2 PZA00486.2 PZA00487.16 PZA00487.24 PZA00492.25 PZA00492.26 PZA00495.3 PZA00495.4 PZA00496.1 PZA00496.2 PZA00497.1 PZA00497.4 PZA00498.3 PZA00499.10 PZA00499.3 PZA00500.5 PZA00501.12 PZA00502.1 PZA00503.5 PZA00504.1 PZA00505.4 PZA00505.5 PZA00506.2 PZA00507.11 PZA00510.2 PZA00510.3 PZA00514.6 PZA00515.10 PZA00516.2 PZA00516.3 PZA00517.4 PZA00517.6 PZA00520.4 PZA00521.3 PZA00522.2 PZA00522.7 PZA00523.2 PZA00524.2 PZA00525.16 PZA00525.2 PZA00527.5 PZA00527.6 PZA00531.1 PZA00531.2 PZA00533.3 PZA00533.4 PZA00534.2 PZA00536.2 PZA00537.4 PZA00538.7 PZA00540.3 PZA00543.2 PZA00543.4 PZA00545.4 PZA00547.6 PZA00547.8 PZA00548.13 PZA00548.3 PZA00549.3 PZA00551.4 PZA00552.3 PZA00552.4 PZA00560.1 PZA00560.2 PZA00562.3 PZA00562.4 PZA00566.5 PZA00567.10 PZA00568.19 PZA00569.11 PZA00569.17 PZA00570.11 PZA00570.19 PZA00571.1 PZA00571.2 PZA00572.1 PZA00572.2 PZA00573.11 PZA00573.3 PZA00578.1 PZA00578.2 PZA00579.2 PZA00579.6 PZA00582.1 PZA00582.4 PZA00583.17 PZA00583.4 PZA00586.1 PZA00587.3 PZA00588.1 PZA00588.2 PZA00589.8 PZA00589.9 PZA00593.2 PZA00595.3 PZA00595.4 PZA00599.25 PZA00603.1 PZA00606.3 PZA00608.1 PZA00610.15 PZA00610.9 PZA00611.1 PZA00613.20 PZA00613.21 PZA00614.1 PZA00614.12 PZA00615.3 PZA00615.6 PZA00616.7 PZA00617.16 PZA00617.5 PZA00618.21 PZA00620.2 PZA00620.3 PZA00621.2 PZA00623.1 PZA00623.2 PZA00626.3 PZA00626.4 PZA00627.1 PZA00628.7 PZA00629.21 PZA00632.1 PZA00633.4 PZA00633.8 PZA00635.1 PZA00635.4 PZA00636.5 PZA00636.6 PZA00637.4 PZA00641.7 PZA00641.8 PZA00644.11 PZA00645.1 PZA00647.9 PZA00650.8 PZA00652.14 PZA00653.5 PZA00654.10 PZA00654.12 PZA00655.1 PZA00658.19 PZA00660.20 PZA00661.6 PZA00662.3 PZA00663.5 PZA00665.6 PZA00667.1 PZA00667.2 PZA00670.2 PZA00672.6 PZA00674.3 PZA00674.4 PZA00676.2 PZA00678.1 PZA00678.2 PZA00680.1 PZA00680.3 PZA00682.16 PZA00682.2 PZA00683.1 PZA00683.2 PZA00684.12 PZA00686.4 PZA00686.7 PZA00693.3 PZA00695.1 PZA00695.2 PZA00697.16 PZA00698.4 PZA00699.1 PZA00699.5 PZA00700.1 PZA00700.3 PZA00701.6 PZA00702.5 PZA00703.1 PZA00704.1 PZA00706.11 PZA00706.16 PZA00707.7 PZA00709.19 PZA00710.1 PZA00710.11 PZA00712.15 PZA00712.4 PZA00714.1 PZA00714.6 PZA00715.1 PZA00715.3 PZA00719.2 PZA00721.4 PZA00721.5 PZA00725.1 PZA00725.4 PZA00726.6 PZA00726.7 PZA00727.1 PZA00727.11 PZA00729.18 PZA00729.9 PZA00730.2 PZA00731.6 PZA00731.7 PZA00732.2 PZA00732.5 PZA02788.12 PZA02789.22 PZA02789.31 PZA02789.36 PZA02791.3 PZA02791.6 PZA02792.16 PZA02792.18 PZA02792.9 PZA02793.33 PZA02806.4 PZA02807.5 PZA02808.12 PZA02808.14 PZA02808.16 PZA02811.4 PZA02811.5 PZA02812.34 PZA02812.43 PZA02814.2 PZA02815.25 PZA02817.15 PZA02817.3 PZA02818.10 PZA02818.6 PZA02819.22 PZA02819.35 PZA02820.16 PZA02820.6 PZA02820.7 PZA02821.5 PZA02824.1 PZA02824.3 PZA02825.4 PZA02830.8 PZA02831.2 PZA02831.6 PZA02833.6 PZA02834.38 PZA02836.17 PZA02837.5 PZA02837.6 PZA02841.3 PZA02841.5 PZA02844.1 PZA02847.21 PZA02850.18 PZA02850.4 PZA02853.10 PZA02853.7 PZA02854.13 PZA02854.7 PZA02855.14 PZA02855.17 PZA02855.22 PZA02861.12 PZA02861.13 PZA02862.10 PZA02862.3 PZA02862.9 PZA02865.11 PZA02868.11 PZA02868.9 PZA02869.2 PZA02869.4 PZA02869.8 PZA02876.10 PZA02878.12 PZA02881.9 PZA02887.9 PZA02888.3 PZA02890.3 PZA02890.4 PZA02890.5 PZA02894.1 PZA02897.12 PZA02906.11 PZA02906.7 PZA02916.4 PZA02916.5 PZA02921.4 PZA02921.9 PZA02923.7 PZA02927.1 PZA02938.3 PZA02938.5 PZA02939.5 PZA02939.6 PZA02941.3 PZA02941.6 PZA02944.10 PZA02944.12 PZA02945.10 PZA02945.37 PZA02946.8 PZA02948.19 PZA02948.21 PZA02949.22 PZA02952.10 PZA02952.7 PZA02955.3 PZA02957.4 PZA02958.17 PZA02959.11 PZA02959.7 PZA02961.1 PZA02961.6 PZA02962.13 PZA02962.14 PZA02963.4 PZA02963.5 PZA02964.7 PZA02965.13 PZA02966.10 PZA02966.11 PZA02968.4 PZA02969.1 PZA02969.8 PZA02970.6 PZA02970.9 PZA02977.4 PZA02981.2 PZA02982.5 PZA02982.6 PZA02983.38 PZA02984.10 PZA02984.7 PZA02985.5 PZA02988.2 PZA02989.5 PZA02992.1 PZA02992.8 PZA02993.14 PZA02993.5 PZA02993.6 PZA02996.9 PZA02997.16 PZA03001.15 PZA03001.18 PZA03001.9 PZA03003.10 PZA03003.11 PZA03004.10 PZA03004.2 PZA03005.19 PZA03005.9 PZA03008.21 PZA03009.5 PZA03009.6 PZA03009.7 PZA03011.6 PZA03012.10 PZA03012.12 PZA03012.7 PZA03013.7 PZA03013.8 PZA03014.21 PZA03014.9 PZA03017.10 PZA03018.16 PZA03018.9 PZA03019.8 PZA03020.8 PZA03024.16 PZA03024.7 PZA03027.12 PZA03027.23 PZA03027.25 PZA03028.5 PZA03032.16 PZA03032.17 PZA03035.5 PZA03036.23 PZA03036.6 PZA03037.2 PZA03037.3 PZA03037.8 PZA03041.8 PZA03042.1 PZA03042.5 PZA03043.14 PZA03043.8 PZA03046.2 PZA03046.3 PZA03046.4 PZA03047.12 PZA03047.20 PZA03048.16 PZA03048.17 PZA03049.14 PZA03049.15 PZA03049.23 PZA03050.11 PZA03052.15 PZA03054.10 PZA03054.3 PZA03054.5 PZA03058.12 PZA03058.16 PZA03058.17 PZA03061.5 PZA03062.15 PZA03062.7 PZA03063.17 PZA03063.18 PZA03064.6 PZA03067.17 PZA03067.20 PZA03068.11 PZA03068.13 PZA03069.4 PZA03069.6 PZA03070.5 PZA03070.6 PZA03070.7 PZA03071.15 PZA03073.23 PZA03073.24 PZA03073.25 PZA03074.15 PZA03074.24 PZA03076.10 PZA03077.13 PZA03078.29 PZA03078.33 PZA03081.1 PZA03081.9 PZA03082.1 PZA03083.7 PZA03085.30 PZA03086.6 PZA03089.12 PZA03090.31 PZA03092.13 PZA03092.7 PZA03093.10 PZA03094.17 PZA03094.6 PZA03094.9 PZA03097.4 PZA03097.7 PZA03100.4 PZA03102.2 PZA03102.9
